# Characterization and Genetic Analyses of New Genes Coding for NOD2 Interacting Proteins

**DOI:** 10.1371/journal.pone.0165420

**Published:** 2016-11-03

**Authors:** Raphaële Thiébaut, Sophie Esmiol, Patrick Lecine, Batoul Mahfouz, Aurelie Hermant, Cendrine Nicoletti, Stephane Parnis, Julie Perroy, Jean-Paul Borg, Leigh Pascoe, Jean-Pierre Hugot, Vincent Ollendorff

**Affiliations:** 1 INRA, UMR866, DMEM, Université de Montpellier, Montpellier, France; 2 UMR1149, INSERM et Université Paris Diderot-Sorbonne Paris-Cité, 75018, Paris, France; 3 Assistance Publique Hôpitaux de Paris, service de gastroentérologie pédiatrique, Hôpital Robert Debré, 75019, Paris, France; 4 Aix Marseille Univ, CNRS, INSERM, Institut Paoli-Calmettes, CRCM, "Cell Polarity, Cell signaling and Cancer - Equipe labellisée Ligue Contre le Cancer", Marseille, France; 5 Aix Marseille Université, Centrale Marseille, CNRS, ISM2 UMR7313, 13397, Marseille, France; 6 CNRS, UMR-5203, Institut de Génomique Fonctionnelle, Montpellier, F-34094, France; 7 INSERM, U1191, Montpellier, F-34094, France; 8 Université de Montpellier, UMR-5203, Montpellier, F-34094, France; 9 Fondation Jean Dausset, CEPH, Paris, France; McGill University, CANADA

## Abstract

NOD2 contributes to the innate immune response and to the homeostasis of the intestinal mucosa. In response to its bacterial ligand, NOD2 interacts with RICK and activates the NF-κB and MAPK pathways, inducing gene transcription and synthesis of proteins required to initiate a balanced immune response. Mutations in NOD2 have been associated with an increased risk of Crohn’s Disease (CD), a disabling inflammatory bowel disease (IBD). Because NOD2 signaling plays a key role in CD, it is important to further characterize the network of protein interacting with NOD2. Using yeast two hybrid (Y2H) screens, we identified new NOD2 interacting proteins (NIP). The primary interaction was confirmed by coimmunoprecipitation and/or bioluminescence resonance energy transfer (BRET) experiments for 11 of these proteins (ANKHD1, CHMP5, SDCCAG3, TRIM41, LDOC1, PPP1R12C, DOCK7, VIM, KRT15, PPP2R3B, and C10Orf67). These proteins are involved in diverse functions, including endosomal sorting complexes required for transport (ESCRT), cytoskeletal architecture and signaling regulation. Additionally, we show that the interaction of 8 NIPs is compromised with the 3 main CD associated NOD2 mutants (R702W, G908R and 1007fs). Furthermore, to determine whether these NOD2 protein partners could be encoded by IBD susceptibility genes, a transmission disequilibrium test (TDT) was performed on 101 single nucleotide polymorphisms (SNPs) and the main corresponding haplotypes in genes coding for 15 NIPs using a set of 343 IBD families with 556 patients. Overall this work did not increase the number of IBD susceptibility genes but extends the NOD2 protein interaction network and suggests that NOD2 interactome and signaling depend upon the NOD2 mutation profile in CD.

## Introduction

Toll Like Receptors (TLR) and Nod-Like Receptors (NLR) are major receptors of the innate immune system [1]. These phylogenetically conserved receptors are widely expressed in epithelial cells, as well as in antigen presenting cells, and they orchestrate the initial immune response toward micro-organisms. TLRs and NLRs recognize common motifs present in bacteria and viruses with some specificities and complementarities allowing the detection, sampling and coordinated response to microbiological insults. NOD2 belongs to the NLR family and this protein is essential to control inflammation of the intestinal mucosa in permanent contact with commensal bacteria [1, 2]. After binding to its peptidoglycan derived MDP ligand, NOD2 and RICK will interact strongly leading to TAK1 recruitment. The resulting multiprotein complex activates the NF-κB and MAPK signaling pathways and subsequently stimulates gene transcription required to initiate the innate immune response. In addition, NOD2 induces autophagy, caspase1 activation, IL-1β secretion and modulates TLR2 signaling [3–7]. All of these functions are part of an efficient immune response, which is crucial for maintaining host homeostasis.

Crohn Disease (CD), a life-long inflammatory condition of the digestive tract which most often occurs in young adults of developed countries [8]. It is a complex genetic disorder involving multiple genetic factors and still undiscovered environmental ones. Up to date, more than 140 susceptibility genes have been identified in large genome-wide association studies (GWAS) [9–17]. Among them, *NOD2* is the main CD susceptibility gene. Up to 50% of Caucasian CD patients carry mutations in *NOD2* [18]. Because NOD2 signaling plays a key role in CD, we hypothesized that genes encoding for NOD2 interacting proteins (NIP) could be sources of additional susceptibility and could explain the disease mechanisms in mutated patients. This hypothesis is strengthened by the discovery that ATG16L1, a NIP involved in autophagy, is a susceptibility gene for CD [3, 4, 12]. Other recently characterized NIPs, such as VIM and TLE1 also appeared to be encoded by CD susceptibility genes [19, 20].

Several other NIPs have already been characterized in regulating either NF-κB activation or alternative NOD2 functions [21]. For instance, RAC1, Erbin, and Centaurin B1 negatively regulate the NOD2 dependent NF-κB pathway, in part by sequestering NOD2 at the plasma membrane [22–25] whereas TRIM27 targets NOD2 ubiquitylation leading to its degradation [26]. Two other NIPs, GRIM19 and hnRNAP1 contribute to bacterial clearance in epithelial cells and to the transcription of the anti-inflammatory IL-10, respectively [27, 28].

Because a complete interaction map of NOD2 would help in delineating the molecular complexity of NOD2 functions, we searched for new proteins interacting physically with NOD2. The genes encoding the identified proteins were also studied in a cohort of European IBD families to assess their role in IBD predisposition.

## Material and Methods

### Patients

A European consortium of gastroenterologists recruited 823 IBD families from Denmark, France, Ireland, Spain and Sweden between January 1997 and December 2000. The diagnoses of CD, UC and indeterminate colitis (IC) were based on Lennard-Jones criteria according to classic clinical, endoscopic, radiological and histopathological findings [29]. The study was approved by the French National Ethic committee (Hospital Saint Louis, Paris, France) and the relevant ethic committees in each country. All participants provided a written informed consent. The 823 IBD families were randomly divided in two cohorts. The first group was formed by 343 IBD families with a total of 556 patients with IBD. The replication cohort contained 467 IBD families with a total of 660 patients with IBD. The IBD families in cohort 1 were composed of 182 CD families containing only healthy individuals and CD patients (277 CD patients), 59 UC families containing only healthy individuals and UC patients (95 UC patients) and 102 families with both CD and UC cases and/or indeterminate colitis (for a total of 184 IBD patients) among family members. The replication cohort was composed of 285 CD only families (389 CD patients), 91 UC only families (126 UC patients) and 91 mixed families (145 IBD patients). The families were previously genotyped for the three main *NOD2* mutations, R702W, G908R and L1007fs and the CD families were thus divided in two subgroups, on one hand the *NOD2* mutated CD families where at least one *NOD2* mutation was segregating and on the other hand the *NOD2* wild type CD families with no *NOD2* mutations. The number of *NOD2* mutated and *NOD2* wild-type CD families were respectively 104 (149 patients) and 78 (128 patients) in the first cohort and 167 (233 patients) and 118 (156 patients) in the replication cohort.

### SNP genotyping

The haplotype structure of the 15 genes was determined according to the HapMap CEU database using the Haploview software [30]. For each gene, SNPs were chosen to have the majority of the haplotypes defined. The SNPs were genotyped in collaboration with the French Centre National de Génotypage by the Illumina or Taqman technologies (Applied Biosystems) [31]. The success rate of this genotyping method was more than 99%. Hardy-Weinberg equilibrium was assessed for all the genotyped SNPs.

### Statistical analyses

TDT was performed in the following family subgroups: IBD, UC, CD, NOD2 mutated CD, NOD2 wild-type CD. TDT was performed for each individual SNP and for the main haplotypes of each gene using the TDT command implemented in the GENEHUNTER package. P values of the tests were corrected for multitesting by the Bonferroni method taking into account the number of tested haplotypes for each gene. A corrected P value lower than 0.05 was retained for significance.

### Yeast two-hybrid screening

MaV203 yeast strain expressing the bait as a fusion with the Gal4 DNA binding domain [32] was transformed with 100 μg of a lung or colon cDNA library cloned in pAD, (Proquest cDNA library, Invitrogen) by the lithium acetate method as described by Walhout, A.J., and Vidal, M [33]. Transformed yeast cells were plated on selective medium supplemented with 20 mM of 3-Amino-1, 2, 4-triazole (3-AT) and incubated for four to five days at 30°C. Positive colonies were picked and patched onto SC-Leu-Trp plates in a 96-well format and grown for 3 to 4 days at 30°C. Growing colonies were then tested by 2 phenotypic assays (β-Gal, Ura+). Plasmids were isolated from clones that were positive for at least two phenotypic markers by incubating yeast cells for 5 minutes at 37°C and 94°C in lysis buffer consisting of 2.5 mg/ml zymolyase (MP Biomedicals) in 0.1 M sodium phosphate buffer pH7.4. The inserts were then amplified by PCR using the Platinium High Fidelity Taq DNA Polymerase (Invitrogen) and the following primers: 5’CGCGTTTGGAATCACTACAGGG3’ and 5’GGAGACTTGACCAAACCTCTGGCG3’. Positive interactions identified were confirmed by the gap repair protocol [34]. Briefly, fresh DB-bait expressing yeast cells were transformed with PCR products corresponding to the selected AD-prey clones and linearized pAD vector. Homologous recombination within yeast allows the reconstruction of the AD-prey plasmids. Recombinant AD-prey plasmids were then selected directly on SC-Leu-Trp plates and phenotypic assays were performed as described above. PCR products from positive clones following Gap Repair experiments were sequenced.

### Expression vectors

Full length cDNAs encoding NOD2 interacting proteins were purchased at the RZPD consortium (Berlin, Germany) or at NITE (Chiba, Japan) and subcloned following PCR in pDONR (Zeo) (Invitrogen) to allow further cloning by recombination in different destination vectors. All cDNAs were fully sequenced. For BRET experiment, cDNAs were cloned into vectors allowing fusion with the donor Renilla luciferase protein or with the EYFP protein (a kind gift of Tarik Issad, France). These vectors were previously modified to facilitate cloning by insertion of a Gateway^™^ cassette (Invitrogen Corporation) allowing N-terminus or C-terminus fusion proteins. Similarly the same gateway cassette was introduced in the pEGFP-C1 and pRK5-myc vectors [35] to express N-terminus EGFP or Myc tagged NOD2, RICK and other proteins.

### Immunoprecipitation experiments

Following 24 h to 48h of transfection (Lipofectamine 2000, Invitrogen) of 3μg of expression plasmid(s), 100 mm diameter plates of HEK293T cells were washed in cold PBS, lysed in 1ml of TX100 lysis buffer containing Tris 50mM PH:7.5, NaCl 150 mM 1% of Triton X100 and a complete protease inhibitor cocktail (Roche). Immunoprecipitations were performed using a monoclonal anti-MYC antibody (9E10) cross linked to agarose beads (9E10 sc-40, Santa Cruz Biotechnology), or a monoclonal anti-GFP antibody recognizing EGFP or EYFP (Roche Applied Science) in the presence of Protein G-Agarose beads (Roche). Immunoblots were revealed with anti-GFP and anti-myc (9E10) monoclonal antibodies.

### BRET analyses

BRET titration experiments were performed by fixing the amount of the donor protein (fused to renilla Luciferase) and by increasing the amount of the acceptor protein fused to enhanced yellow fluorescent protein (EYFP) that was coexpressed by transient transfection in HEK293T cells distributed in 6 wells plates. Transfections were performed using JET PEI (4μl/μg of DNA, Polyplus) with a total of 1μg DNA/well each containing for instance, 300 ng of a plasmid encoding donor protein (for example pLucRICK) and increasing amount of the acceptor pEYFPNOD2 plasmid (0 ng, 50 ng, 100 ng, 200 ng, 400 ng or 800 ng). pBluescript was used to normalize DNA amount to 1μg. 24h after transfection, cells were sometimes stimulated with the NOD2 ligand MDP 10μg/ml during 18h to 22h. To measure BRET signal, cells were collected 48 hours after transfection, and resuspended in 300μl of PBS containing 0,1% of glucose, deposed in triplicate in a white 96 wells microplate. Coelenterazine H (interchim) was added to each well (final concentration 5μM). BRET signal is measured over a 20 minutes period with a Biotek synergy2 reader that allows the sequential detection of the emission signal at 530 and 480nm. BRET signal is then calculated by determining the emission ratio 530/480 and by subtracting the background 530/480 ratio of cells expressing only donor proteins. Following Coelenterazine H hydrolysis, the luciferase fused donor protein emits light in a spectrum range allowing excitation of the EYFP acceptor protein and BRET signal. To evaluate the level of each expressed donor protein, total luminescence is measured by calculating the mean of the triplicate initial reading at 480nm immediately following Coelenterazine H addition. Similarly total fluorescence is measured to quantify the level of each expressed acceptor protein fused to fluorescent EYFP following excitation at 485nm and reading at 530nm.

### Small Interfering RNAs (siRNAs) and NF-kappaB reporter assays

The ON-TARGETplus SMARTpool siRNAs targeting RICK, ANKHD1, LDOC1, CHMP5, DOCK7, PPP1R12C, PPP2R3B, SDCCAG3, or TRIM41 and a non-targeting control were purchased from Dharmacon (Lafayette, CO). HEK293T cells were transfected with siRNAs at a final concentration of 50 nM, using DharmaFECT1 (Dharmacon) in 6 wells plates. One day after, HEK293T cells were split at 10^5^ cells/well in 24 well plates. Cells were transfected the following day with a NFκB Luciferase reporter construct (Cignal, SABiosciences) and 0,2 ng of plasmid expressing mycNOD2 or empty vector, using Lipofectamine 2000 (Invitrogen). The following day, cells were stimulated or not with 10μg/ml MDP during 6h and dual reporter measurements were carried out as recommended by manufacturer’s instruction (Promega). Specificity and efficiency of each siRNA was verified by RTQPCR analysis using validated primers (SABiosciences) on RNA made 48h hours after siRNA transfection in parallel transfection experiments. 70 percent or more of target mRNA inhibition was considered as a successful siRNA experiment.

### RTQPCR arrays analyses

RNA was extracted 24h after treatment with MDP and/or LPS with the Mascherey-Nagel RNAXS kit. Customized PCR arrays (SABiosciences) were performed and analyzed by TEBU-BIO (France). Two housekeeping genes, GAPDH and TBP were used to analyse the expression rate of the genes of interest and the ΔΔCt method was applied.

## Results

### NOD2 interactome

**Fig 1:**
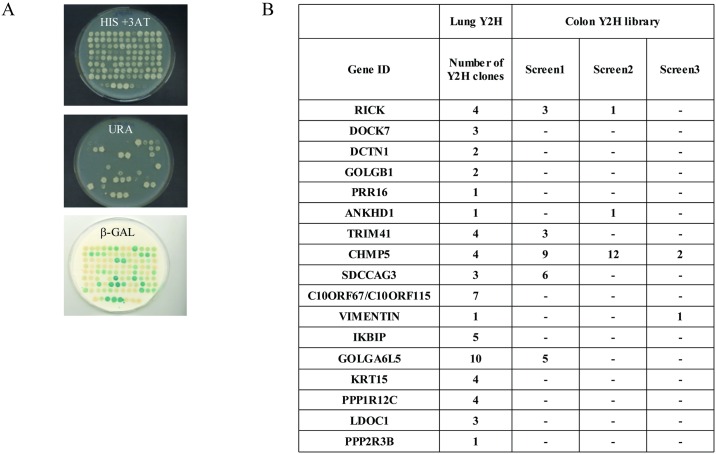
Yeast two hybrid screens using NOD2 as a bait. (A) Colon and Lung cDNA libraries fused to GAL4AD were screened with NOD2 full length protein fused to GAL4DBD and transformed yeast cells were plated onto minimal selective medium lacking Tryptophan, Leucine and Histidine supplemented with 20 mM of 3-Amino-1,2,4-triazole (3-AT) and incubated for four to five days at 30°C. Positive colonies were picked and patched onto SC-Leu-Trp plates in a 96-well format and grown for 3 to 4 days at 30°C. A) Growing colonies were tested by 3 phenotypic assays (His+ 3-AT, Ura+, β-Gal). (B) Colon and lung Y2H preys isolated with NOD2 as a bait. Number indicates how many clones were identified in each Y2H screen.

In order to identify new NOD2 interacting proteins, extensive Yeast 2-Hybrid (Y2H) screens were performed using a modified version of the Y2H system that reduces the rate of false positive clones [33, 36]. Yeast strain Mav 203 with three integrated reporter genes was used to isolate NIPs by screening a colon and a lung cDNA library with a human *NOD2* full length cDNA as a bait. Positive clones were then isolated and their phenotypes on medium lacking Uracile or histidine, as well as their β-Galactosidase activity, were assessed (Fig 1 and S1 Table). To confirm each putative interacting protein isolated in the initial screen, a “gap repair” step was performed (see material and methods) [34]. Finally, 59 clones (encoding 17 different proteins) were obtained in the lung library, and 82 clones (22 proteins) following 3 screens in the colon library (S1 and S2 Tables). Overall, the screen in the lung library gave more transformants and the identified proteins were often isolated more than once. In contrast, more than half of NIPs candidates from the colon cDNA library were isolated only once making them possible false positive (S2 Table).

Several clones coding for RICK protein were identified in the colon or lung library, validating the Y2H screening procedure, since RICK is an undisputed NIP. All RICK positive clones contained the C-terminus CARD domain of RICK, a region known to be involved in a heterotypic interaction with the N-terminus CARD domains of NOD2 [37]. Importantly, these results indicated that the overall conformation of the NOD2 protein is not compromised when fused to GAL4DBD and thus allows isolation of valid partners in Y2H. Several putative NIPs (PPP1R12C, LDOC1, ANKHD1, CHMP5 or IKBIP) were previously reported to be linked with the NF-κB signaling pathway, confirming their interest as potential candidates [30, 38–41]. Others were involved in cellular trafficking, protein sorting or cytoskeletal architecture (CHMP5, DOCK7, VIM, SDCCAG3, KRT15, DCTN1, and GOLGB1) [42–47] or as regulators of cell signaling (PPP2R3B, TRIM41) [48, 49]. For some proteins including C9orf150, PRR16, C10orf67-C10orf115 and GOLGA6L5, no hint about their possible function could be obtained in the literature or inferred by scrutinizing their amino acid sequences. Some NIPs could in fact be Y2H false positives, such as GOLGA6L5, which is considered as a pseudogene not able to generate a full length protein product and this protein was therefore not chosen for further studies [50]. Without eliminating them as relevant NIPs, it is also noteworthy that VIM and KRT15 were reported as false positive in some interaction studies [51].

The NIP candidate CHMP4b was found several times only in the colon library (S2 Table). As CHMP5 (vps60) identified in all four Y2H screens, CHMP4b (vps32) belongs to the ESCRTIII proteins family involved notably in multivesicular bodies (MVB) biogenesis, cellular endosomal signaling, protein sorting and autophagy [52, 53].

As a result of Y2H screens, 14 preys were selected to further investigate their interaction with NOD2. Several proteins like RICK, CHMP5, TRIM41, SDCCAG3, VIM, and ANKHD1 were chosen because they were identified independently in the colon and the lung cDNA library. The other selected proteins (DOCK7, C10orf67, PPP1R12C, LDOC1, KRT15, IKBIP, PPP2R3B and PRR16) included mostly NIP candidates isolated a minimum of 3 times.

### Co-immunoprecipitation experiments

**Fig 2:**
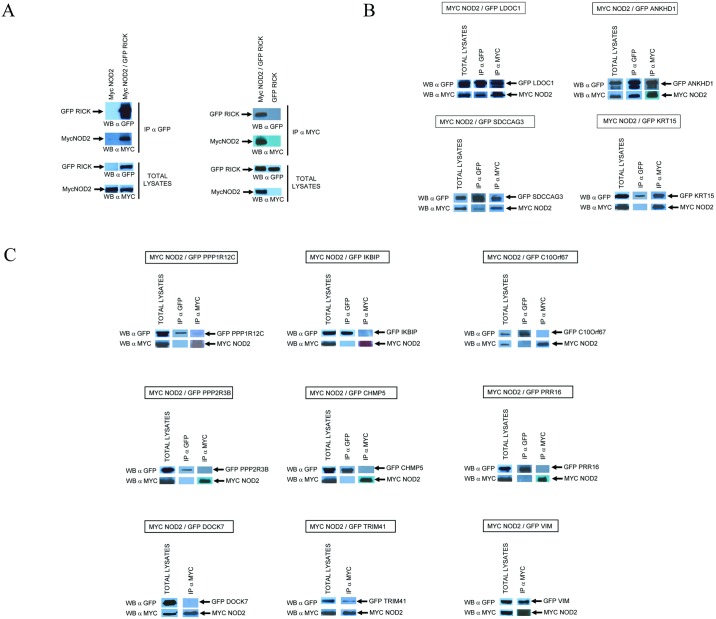
Co-Immunoprecipitations between NOD2 and NIP candidates. NOD2 cDNA was tagged with a MYC epitope at its N-terminus and all other NIP cDNAs were tagged with EGFP. Proteins were co-expressed in HEK293T cells and 24 h after transfection, cells were lysed in a buffer containing 1% TX100. Cell lysates were first analyzed by Western blotting with anti-MYC and anti-GFP antibodies to verify the level of expression and the correct size of each protein (Total lysates). Cell lysates were then subjected to immunoprecipitation with anti-MYC (IP αMYC) and/or anti-GFP (IP αGFP) antibodies and analyzed by Western Blot with αMYC and αGFP. (A) Co-Immunoprecipitation of MycNOD2 and EGFP-RICK after αGFP immunoprecipitation and αMYC Immunoprecipitation. Negative control experiments showed that lysates containing only MycNOD2 proteins could not be immunoprecipitated with αGFP antibodies and reciprocally, lysates containing only GFP-RICK proteins could not be immunoprecipitated by αMYC antibodies (B) Immunoprecipitation experiments between MycNOD2 and 13 NIP candidates. Immunoprecipitation and Western Blot analysis are performed as in A on cell lysates coexpressing MycNOD2 and a specific GFP tagged NIP.

Co-immunoprecipitation experiments were performed to confirm Y2H interactions. The full length (or near full length) cDNAs coding for the 14 selected NIPs including RICK were cloned in a plasmid to express EGFP fused proteins. Each EGFP-tagged NIPs was co-expressed with Myc tagged NOD2 protein in HEK293T cells by transient transfection. The cells were then lysed in a “stringent” buffer containing 1% TX100 and subjected to immunoprecipitation with anti-myc (to immunoprecipitate myc-NOD2) or anti-GFP antibodies (to immunoprecipitate each EGFP-NIP) and immunoprecipitated proteins were analysed by Western blot using anti-myc and anti-GFP antibodies. RICK and NOD2 were used as positive controls (Fig 2A left panel). New NIP candidates co-precipitated with NOD2 either strongly and in both orientations—ie following IP of mycNOD2 and IP of EGFP-NIP, (ANKHD1, LDOC1, SDCCAG3, RICK) or more weakly and/or in only one orientation (PPP1R12C, KRT15, VIM, TRIM41) (Fig 2A right panel and Fig 2B). Six putative NIPs did not co-precipitate with NOD2 in these conditions (DOCK7, PPP2R3B, IKBIP, CHMP5, C10orf67, PRR16) (Fig 2B). This method could however generate false negative results by disrupting some subcellular compartments or a multi-protein complex required for a proper interaction to occur. We thus developed additional assays to confirm and extend Y2H and co-immunoprecipitation experiments.

### BRET

**Fig 3:**
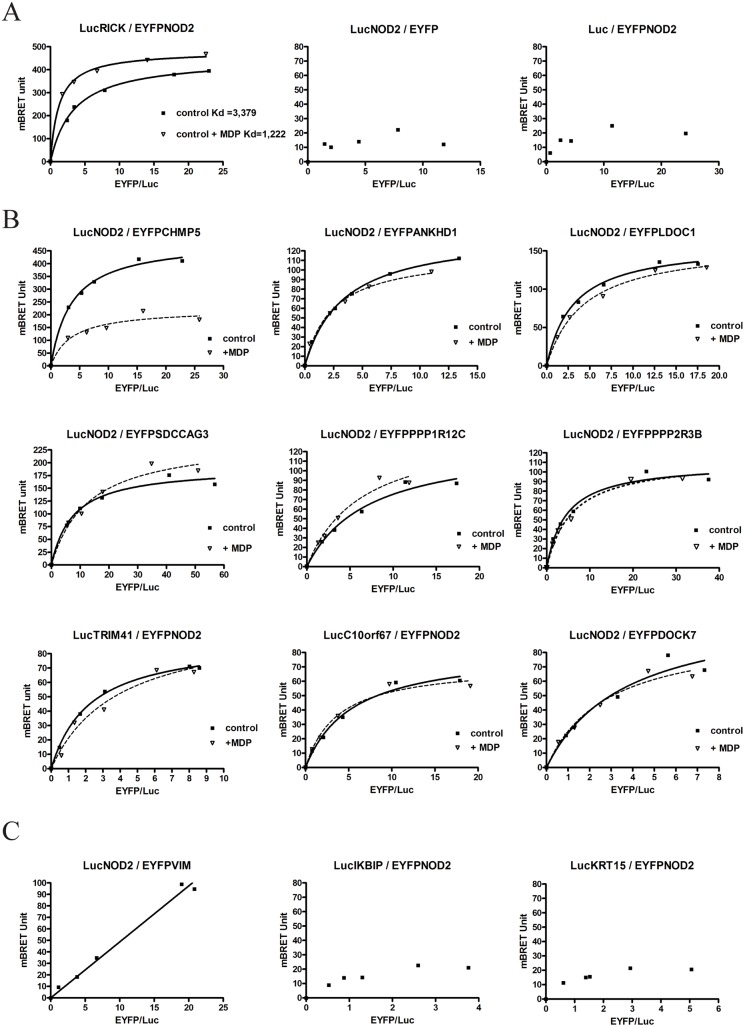
Interaction between NOD2 and NIP by BRET. BRET titration experiments were performed by fixing the amount of the donor protein (fused to renilla Luciferase) and by increasing the amount of the acceptor protein (fused to EYFP) coexpressed by transient transfection in HEK293T cells. A specific energy transfer can be detected when the two proteins interact directly (i.e. within a distance of 10 nm or less) and must increase hyperbolically as a function of the acceptor/donor (Fluorescence/Luminescence) ratio. In comparison, non-specific interactions and random collisions would increase linearly. Regression curves assuming one binding site are represented as BRET value (mBRET unit) as a function of the Fluorescence/Luminescence ratio. In these BRET titration experiments there are no unit for the x-axis since it is a ratio of protein measuring the relative expression of each BRET protein partner (ratio between the acceptor -fused to EYFP and the donor-fused to luciferase). BRET saturation (BRETmax value) is reached when all expressed donor proteins are involved in an interaction with an acceptor protein. BRET experiments only provide a relative affinity for proteins (apparent Kd). The relative affinity between 2 proteins (apparent Kd) corresponds to the BRETmax/2 value (BRET50). Experiments were performed at least 3 times and a representative experiment is shown. All titration regression curves (in absence of MDP) fitted with a R^2^ coefficient >0.98 except for LucNOD2 / EYFPDOCK7 (R^2^ = 0.9621). (A) BRET titration experiments between donor LucRICK and acceptor EYFPNOD2 in absence (control) or in presence of a 22h MDP stimulation (10μg/ml). Negative control BRET experiments were performed with the LucRICK donor replaced by unfusioned Luciferase (middle panel) or with the acceptor EYFPNOD2 replaced by EYFP (right panel). (B) BRET titration experiments between NOD2 and 9 NIP candidates in absence (control) or in presence of MDP for a 18-22h stimulation. (C) BRET titration experiments between NIPs candidates and NOD2 showing no specific BRET saturation signal: Non-specific BRET linear profile for NOD2/VIM (left), and BRET values below threshold (50 mBRET Unit) for NOD2/IKBIP (middle) and NOD2/KRT15 (right).

The Bioluminescent Resonance Energy Transfer (BRET) approach reveals direct interaction between proteins in the cellular context without cell lysis [54]. Following coelenterazine H hydrolysis, energy transfer between a protein A fused to Renilla Luciferase (the donor) and a protein B fused to EYFP (the acceptor) occurs only when the distance between the two proteins of interest is under 10 nm. For this purpose NOD2 cDNA and each putative NIP cDNA were subcloned in the BRET vectors to produce NH2-terminus and COOH-terminus fusions with Renilla luciferase (Rluc) and EYFP. Following transient transfection in HEK293T cells, initial experiments were carried out to select the orientation giving the strongest BRET signal for each interacting candidate (data not shown). As a positive control, the interaction between LucRICK and EYFPNOD2 was measured. Saturation curve analysis showed a hyperbolic profile demonstrating a specific interaction between RICK and NOD2. Incubation with MDP modified this saturation profile and curve analyses indicated an increase of affinity between NOD2 and RICK following MDP stimulation corresponding to a decrease of their apparent Kd (Fig 3A). Negative control BRET experiments were also performed with the LucRICK donor replaced by unfusionned Luc or with the acceptor EYFPNOD2 replaced by the EYFP (Fig 3A). Similarly BRET saturation experiments were carried out for the thirteen new putative NOD2 partners. Most of them gave hyperbolic BRET saturation curves reflecting specific interactions (Fig 3B). Only background BRET signal (BRET value below the threshold of 50 mBRET unit) could be obtained however between NOD2 and KRT15, IKBIP or PRR16 (Fig 3C and data not shown). Moreover, the BRET signal between NOD2 and the VIM protein was considered non-specific, since the experimental curve did not reach a plateau and appeared as a linear rather than a hyperbolic curve (Fig 3C). This non-specific response is more likely due to random collision between donor and acceptor proteins (bystander effect).

In order to understand the impact of NOD2 activation on the NIP-NOD2 interaction, BRET experiments in live cells were also carried out with the addition of MDP following transfection. Among all the proteins investigated, only the CHMP5/NOD2 BRET profile appeared clearly modified by the addition of MDP. In that case, an overnight incubation with MDP induced a decrease in BRETmax of the NOD2/CHMP5 interaction, indicating either a conformational change or dissociation of the protein complex after MDP activation (Fig 3B). This result strongly suggests a direct role of CHMP5 in the NOD2 pathway.

An interaction score was calculated as an attempt to classify the NIPs characterized in this study. This score (arbitrary unit) integrates, the number of hits in Y2H screens, the “strength” of the co-immunoprecipitation and the profile of the BRET saturation curves and MDP response (Table 1). In summary of these interactions studies, 6 NIPs were confirmed by coimmunoprecipitation and BRET (RICK, ANKHD1, LDOC1, SDCCAG3, TRIM41, and PPP1R12C), 6 NIPs were confirmed only by one method (coimmunoprecipitation only for KRT15 and VIM, BRET only for CHMP5, DOCK7, PPP2R3B, and C10orf67), and at last, 2 NIPs were not confirmed by either method (PRR16, IKBIP).

**Table 1:** Summary of Y2H, co-IP and BRET studies.

NIP Gene ID	Y2H	Co-Ip	BRET	Interaction score (Total)
**RICK**	**20 +**	**20**	**20**	**60 +**
**ANKHD1**	**10 +**	**20**	**10**	**40 +**
**CHMP5**	**20 +**	**0**	**20**	**40 +**
**SDCCAG3**	**15 +**	**15**	**10**	**40 +**
**LDOC1**	**10 +**	**20**	**10**	**40 +**
**TRIM41**	**20 +**	**10**	**10**	**40 +**
**PPP1R12C**	**15 +**	**5**	**10**	**30 +**
**KRT15**	**15**	**10**	**0**	**25**
**C10ORF67**^a^	**15**^a^	**0**^a^	**10**^a^	**25**^a^
**VIMENTIN**	**10**	**10**	**0**	**20**
**DOCK7**	**10**	**0**	**10**	**20**
**PPP2R3B**	**5 +**	**0**	**10**	**15 +**
IKBIP	15	0	0	15
PRR16	5	0	0	5

Confirmed NIPs by Co-Immunoprecipitation or BRET are in bold. An interaction score was calculated for each NIP as follows: This interaction score (arbitrary unit) integrates relevant data obtained from Y2H, COIP and BRET experiments. For Y2H (5 to 20 points): 5 points were given for one Y2H hit, 10 points for 1 to 3 hits, 15 points for more than 3 hits; 5 points were added when a particular NIP was isolated in at least 2 independent Y2H screens. A (+) indicates that an interaction with NOD2 was reconfirmed by Y2H using the full length cDNA encoding for a given NIP. For Co-immunoprecipitation (0 to 20 points): 0 for no coimmunoprecipitation, 5 for a weak coimmunoprecipitation, 10 for a clear coimmunoprecipitation in one orientation, 20 for a coimmunoprecipitation in both orientations. For BRET (0 to 20 points): 0 point, when no specific BRET signal could be obtained; 10 points for a specific hyperbolic saturation curve; 20 points when this saturation curve was modified by a MDP stimulation.

^a^Coimmunoprecipitation and BRET experiments were performed with a full C10orf67 cDNA. However the Y2H cDNA hits were chimeric cDNA arising from the fusion between C10orf67 and C10orf115 loci.

### NF-κB reporter assays

**Fig 4:**
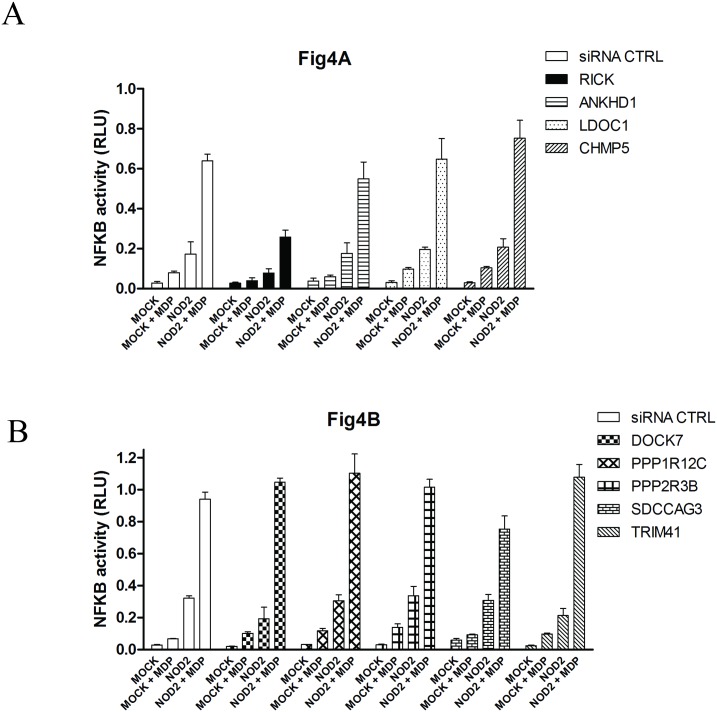
NOD2 dependent NF-κB activity in HEK293T cells following siRNA targeting different NIPs. 48h after siRNA transfection, HEK293T cells were transfected in triplicate with a NF-κB Luciferase reporter plasmid mix containing a NF-κB dependent Firefly Luciferase and a normalisation Renilla luciferase plasmids (SABiosciences) together with 0,2 ng of mycNOD2 expression vector. Luciferase activity was measured 24 h after NOD2 transfection. Cells were stimulated or not with MDP (10 μg/ml) during 6 hours. NF-κB activity is shown as Relative Luciferase Unit (RLU) corresponding to the NF-κB dependent Firefly Luciferase signal divided by the Renilla signal (Normalisation). Error bars correspond to the Standart error of the mean. Experiments were done three times with similar results.

To evaluate the functional role of each NIP on the NOD2 dependent NF-κB signaling, reporter assays were carried out in HEK293T cells transfected with plasmid encoding luciferase under the control of NF-κB response elements. As reported elsewhere, overexpressing NOD2 in these cells stimulates NF-κB and a MDP incubation further increased NF-κB activation (Fig 4). The cells were first transfected either with a non-targeting control siRNA or with a siRNA targeting a specific NIP mRNA. Quantitative RT-PCR assays confirmed that each targeted mRNA was decreased by at least 70% 48h after siRNA transfection (data not shown). As expected the NF-κB response was decreased by transfection with a RICK siRNA, confirming that RICK is required to fully activate NF-κB after NOD2 transfection and MDP stimulation (Fig 4A). In contrast, none of the NIP siRNA had a significant positive or negative effect on NF-κB activation after NOD2 transfection and stimulation by MDP (Fig 4A and 4B). These results indicate that these NIPs do not exert a primary role on NOD2-dependent NF-κB activation in HEK293T cells.

### NIP gene expression in the stimulated THP1 macrophage cell line

Nod2 is constitutively expressed by the THP-1 monocyte cell line. The expression of Nod2 and 10 NIP genes was studied by qRT-PCR arrays upon MDP, LPS or LPS + MDP stimulation (Table 2). Nod2, Vim and Chmp5 were significantly upregulated while Ldoc1 and Trim41 appeared down regulated by a LPS + MDP stimulation (threshold of significance x2). Concerning Chmp5, others studies have reported analogous up-regulation upon LPS or bacterial treatment [55, 56]. The expressions of Ankhd1 and Ppp2r3b were respectively increased and decreased mostly in the presence of MDP + LPS but these changes were not statistically significant. Finally, Dock7, Sdccag3 and Ppp1r12c mRNA expression were not affected by MDP and/or LPS stimulation. Overall these expression data reinforce the link between NOD2 and CHMP5, TRIM41, VIM, LDOC1, ANKHD1 and PPP2R3B.

**Table 2:** Fold regulation by MDP or LPS in THP-1 cell line.

	Fold up or down regulation		
Gene Symbol	After MDP	After LPS	After MDP+LPS
**NOD2**	**17.8**	**4.05**	**24.85**
DOCK7	-1.22	-1.25	-1.08
ANKHD1	1.47	-1.20	1.87
**TRIM41**	**-1.99**	**-2.42**	**-2.03**
**CHMP5**	1.51	1.58	**2.11**
SDCCAG3	-1.07	-1.23	-1.15
**VIM**	**5.95**	**3.10**	**7.16**
IKBIP	1.22	-1.26	1.09
PPP1R12C	-1.08	-1.03	-1.06
**LDOC1**	-1.64	**-3.02**	**-4.23**
PPP2R3B	-1.35	-1.50	-1.75

THP-1 cells were stimulated with 10μg/ml of MDP or 0.01μg/ml of LPS for 24h. ARN were then extracted and analyzed by QPCR array (TEBU-BIO). The experiments were done in duplicate. The results were obtained with the 2-ΔΔCt method with TBP and GAPDH as housekeeping gene and a threshold of 2 fold was considered as a significant change in expression (in bold).

### Interactions between NIPs and main CD associated NOD2 mutants (R702W, G908R and 1007fs)

**Fig 5:**
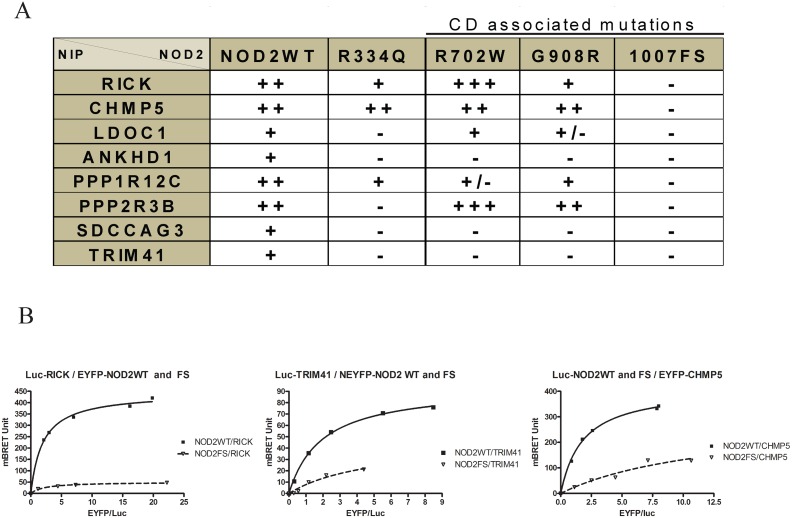
Interaction between NIPs and NOD2 mutants. (A) Interaction between 8 full length NIPs and different NOD2 mutants (Blau or CD associated mutations) were tested and scored by Y2H using three reporter genes (lacZ/HIS3 and URA). (B) BRET titration experiments (as described in Fig 3) compared the interaction of NOD2WT and NOD2FS mutant (1007fs) with RICK, CHMP5 and TRIM41.

The interaction between 8 NIPs and the three main CD associated NOD2 mutants were tested by Y2H. By this analysis (Fig 5A), we observed that none of the NIPs interacts with the NOD2FS mutant (1007fs) and we confirmed by additional BRET titration experiments that the interaction of RICK, CHMP5 and TRIM41 is in fact greatly compromised with this NOD2 mutant (Fig 5B). In contrast, some NIPs (RICK, LDOC1, PPP1R12C, PPP2R3B, and CHMP5) interact with NOD2 CD associated mutants (R702W and G908R) and interestingly, RICK and PPP2R3B appear to bind better to the R702W than to the G908R mutant. Other NIPs (ANKHD1/SDCCAG3/TRIM41) do not interact at all with these CD mutants. These results show that mutation targeting the C-terminus of NOD2 (notably within the LRR repeats) strongly altered its interaction with the NIPs isolated here and this indicates that the position of the mutation in NOD2 can affect NOD2 interactome. Moreover we tested another NOD2 mutant (Blau R334Q) not associated with CD but with Blau syndrome and targeting another region of NOD2 protein named the NACHT domain (Fig 5). Similarly, the interaction of most NIPs tested with this mutant (with the exception of CHMP5) was strongly compromised compared to NOD2WT reinforcing the hypothesis that NOD2 mutation(s) can affect its interaction with NIPs (Fig 5).

### Genetic analyses

Genetic analyses were performed for genes encoding 15 NIPs with a set of 101 Single Nucleotide Polymorphisms (SNP) chosen to cover the vast majority of gene haplotypes with a frequency higher than 0.05, as estimated on the Hapmap website (Table 3). Studies were done on a panel of 343 IBD families for a total of 556 patients IBD. Transmission disequilibrium tests were calculated for the full cohort, for the 182 CD only families (277 CD patients) and for the 59 Ulcerative Colitis (UC) only families (95 UC patients). In addition, for genetic analyses, the group of CD only families was split in two subgroups, depending on the presence or not of a *NOD2* mutation.

**Table 3:** Gene and SNP ID of the new NOD2 partners.

Gene ID	localization	Number of haplotypic bloc	Number of haplotype	Number of haplotypewith Freq>0.05	Number of tagged haplotype with Freq>0.05	SNPs ID
**DOCK7**	**1p31.3**	**1**	**9**	**4**	**4**	**rs10493326; rs1168026; rs11577840; rs13375691; rs17381383; rs4915846; rs11207998; rs7419069; rs12117388; rs912540**
**DCTN1**	**2p13**	**1**	**3**	**2**	**1**	**rs9309484**
**GOLGB1**	**3q13**	**1**	**10**	**8**	**8**	**rs9968051; rs9819530; rs9852845; 6803839; rs11927625; rs10049460; rs10470410; rs1919555; rs1919554; rs12498138**
**PRR16**	**5q23.1**	**13**	**63**	**42**	**28**	**rs7715549; rs4895255; rs2601208; rs2601209; rs2655069; rs2655066; rs2691100; rs7710848; rs921782; rs4895160; rs1159767; rs1524558; rs9327147; rs12523269; rs6862090; rs13161840; rs300974; rs300970; rs13174421; rs2077726; rs11241254; rs17427447; rs6865072; rs1524565; rs1375462; rs1584465; rs985263; rs12514071; rs12519395; rs10042938; rs2218717; rs17428689; rs1466107; rs1449142; rs7736461; rs716815; rs12187844; rs10519647; rs4895272**
**ANKHD1**	**5q31.3**	**1**	**9**	**8**	**1**	**rs1432959; rs10042299; rs3733681; rs1835959; rs9324644**
**TRIM41**	**5q35.3**	**1**	**6**	**5**	**3**	**rs2545098; rs7727787; rs2770957; rs2261114**
**CHMP5**	**9p13.3**	**2**	**5**	**5**	**5**	**rs831271; rs831276; rs831275**
**SDCCAG3**	**9q34.3**	**3**	**15**	**9**	**9**	**rs1132005; rs3812578; rs10706; rs4298601; (rs10870446)**
**C10orf67**	**10p12.2**	**3**	**10**	**8**	**8**	**rs10828426; rs11013389; rs4259746; rs10828431; rs2036921**
**VIM**	**10p13**	**1**	**8**	**5**	**3**	**rs3758413; rs7914640; rs11254468; rs1980662**
**IKBIP**	**12q23.1**	**2**	**11**	**11**	**6**	**rs1048906; rs1055656; rs17028572; rs4762501; rs12371097; rs1281083**
**KRT15**	**17q21.2**	**1**	**5**	**3**	**3**	**rs2662; rs2305556; rs3744784; rs3760519**
**PPP1R12C**	**19q13.42**	**1**	**6**	**5**	**5**	**rs7259963; rs604216**
**LDOC1**	**Xq27**	**1**	**7**	**3**	**3**	**rs710106; rs4824993**
**PPP2R3B**	**Xp22.33; Yp11.3**	**2**	**9**	**7**	**1**	**rs2738319**

The frequency used to for each haplotype was determined according to the Haploview software from HapMap CEU population.

For each gene, TDT (transmission disequilibrium test) was tested for each SNP independently and for all possible haplotypes with a combination of 2 or 3 of the genotyped SNPs in the different family groups. Among the 15 genes, we found a weak distortion of transmission for haplotypes in 5 NIPs: *DOCK7*, *GOLGB1*, *IKBIP*, *PRR16*, and *VIM* (Tables 4 and 5).

**Table 4:** TDT results for DOCK7, GOLGB1 and IKBIP for haplotypes with frequency >0.05.

Gene ID	Haplotype frequency^1^	Phenotype	SNP ID^2^	T/U	p-value
DOCK7	0.142	CD	rs17381383	55/31	0.0097
GOLGB1	0.275	CARD 15 mutated CD	rs9852845 (MAF);rs6803839 (RAF); rs10470410(RAF)	51/27	0.0065
GOLGB1	0.058	CD	rs12498138 (RAF); rs9852845(MAF)	24/9	0.0090
IKBIP	0.096	CARD 15 non mutated CD	rs17028572(MAF); rs4762501(RAF); rs12371097(MAF)	24/9	0.0090
IKBIP	0.20	UC	rs4762501(MAF); rs12371097(RAF)	25/7	0.0014
IKBIP	0.20	UC	rs17028572(RAF); rs12821083(RAF)	23/6	0.0016

Transmission disequilibrium test for major haplotypes (frequency>0.05).

^1^: Haplotype frequency in HapMap CEU panel.

^2^: SNP ID defining the haplotype considered.

RAF: Rare allele frequency and MAF: major allele frequency of the considered SNP. T: transmitted; U: Untransmitted.

**Table 5:** *VIM* results for haplotypes with frequency >0.05.

			1^st^ cohort		2nd cohort	
Haplotype Frequency	SNP ID	Phenotype	T/U	p-value	T/U	p-value
0.557	rs11254468 (MAF); rs1918662 (MAF)	CARD15 WT CD	30/9	0.0008	-	-
0.257	rs11254468 (RAF)	CARD15 WT CD	-	-	35/19	0.0290

Transmission disequilibrium test for major haplotypes (frequency>0.05). RAF: Rare allele frequency and MAF: major allele frequency of the considered SNP. T: transmitted; U: Untransmitted

For *DOCK7*, a positive TDT was seen for rs17381383 in the group of CD only families (minor allele frequency: T: 55 U: 31 p = 0.01). This marker tags a haplotype with a frequency of 0.142 in the Hapmap CEU panel and 0.15 in our cohort (Table 5). For *GOLGB1*, a distortion of transmission was observed for an haplotype with a frequency in HapMap CEU panel of 0.275 in *NOD2* mutated CD families (T: 51 U: 27 p = 0.007) and for another haplotype in the whole group of CD families (T: 24 U: 9 p = 0.0090) (Table 4). A nominal positive TDT were found for an haplotype with a frequency of 0.20 in the UC only families (T: 25 U: 7 p = 0.002 and T: 23 U: 6 p = 0.002) and for an haplotype with a frequency of 0.096 in the NOD2 wild-type CD families (T: 24 U: 9 p = 0.009, Table 4). *PRR16* is a 222.9kbp gene with a highly complex haplotypic structure defined by at least 13 haplotypic blocs. 39 markers were used to analyze the gene. Several positive TDT (p>0.001) were found in the IBD family cohort most often with haplotypes containing of the intronic SNP rs13161840. Similarly, distortions of transmission were observed in the *NOD2* mutated CD families with haplotypes containing the minor frequency allele of rs300970. However, the p values of all these TDT did not remain significant after applying the Bonferoni correction for multitesting.

*VIM* is defined by one haplotypic block and 8 haplotypes. The 4 genotyped SNPs were available defining only 3 different haplotypic groups in the 8 haplotypic group defining VIM in HapMap CEU panel. A distortion of transmission was observed for an haplotype with a frequency of 0.557 in NOD2 WT CD families (T: 30, U: 9; p = 0.0008, Pc<0.005 (Table 5). Because the test remained significant after Bonferoni correction, we genotyped a replication cohort formed by 467 (660 affected family members) independent IBD families. In this second cohort, another weak distortion of transmission was found, but for another haplotype with a frequency of 0.257 in NOD2 wild-type families (T:35 U:19; p = 0.029) (Table 5). As the two cohorts are believed to have similar genetic profiles, and a different haplotype was preferentially transmitted in each cohort, the transmission distortion could not be considered to be replicated.

## Discussion

The main goal of this work was to gather proteomic and genetic data focusing on novel proteins interacting with NOD2, a central actor of the intestinal innate immune response. Because NOD2 is the main CD predisposing gene, we also tested whether the genes encoding these proteins could represent new IBD susceptibility genes.

In this work we analyze numerous positive hits obtained by Y2H screens using NOD2 as bait. In contrast to previous screens with NOD2 [25, 27], we used a full-length protein rather than a truncated version of NOD2. Indeed, the candidate proteins isolated in our study with the full-length protein did not interact with individual separate domains of NOD2 (CARDs, NACHT, LRR), with the exception of RICK interacting with the NOD2 CARDs (data not shown). This observation suggests that the 3D conformation of NOD2 may be important, as distinct from the simple juxtaposition of its three main domains. It also implies that previous Y2H screens using a truncated NOD2 protein may have underestimated the number of positive clones and thus the number of interacting proteins [25, 27].

We have established by Y2H, coimmunoprecipitation and BRET methods that NOD2 can interact with numerous previously uncharacterized proteins expressed in human tissues (colon and lung). These novel protein partners extend the NOD2 interactome and provide new leads about mechanisms of NOD2 function, regulation, and subcellular localization. The use of two independent methods to confirm Y2H interactions allowed validation of a total of eleven NOD2 interacting candidates. Importantly, some interactions could not be seen by co-immunoprecipitation, but were positive in BRET experiments, suggesting that the intracellular milieu and/or the subcellular localization can be important for some specific interactions to occur.

Based on a generic NF-κB reporter assay, combined with the use of siRNA, none of the NIPs isolated here, except RICK, appear to be major regulators of the NOD2 dependent NF-κB pathway at least in HEK293T cells. These data are however consistent with a recent publication reporting the identification by genome wide RNAi screen in HEK293 cells of new positive and negative regulators of the NOD2 dependent NF-κB pathway [57]. Of note, in this study WBP11 has been found as a positive regulator of NOD2-dependant NF-κB activation and to interact with NOD2 by pulldown. We have also identified WBP11 as a NOD2 partner by one colon Y2H screen (S2 Table) [57].

The expression of several NIPs is upregulated by MDP and LPS in THP-1 cells suggesting that these proteins may play a role as feedback control to amplify or down regulate the NF-κB signaling pathway. Alternatively some NIPs may be involved in other specific NOD2 functions including autophagy induction, Interleukin production (IL-8 or IL1β) or in the subcellular localization and traffic of NOD2 during signaling.

Importantly, our data indicate that the 3 main NOD2 mutants associated with CD, R702W, G908R and 1007fs (representing more than 80% of all NOD2 mutations found in CD patients) interact differentially with 8 NIPs and this could have functional consequences in term of downstream signaling. Future studies will help define whether these alterations (loss or gain) of interactions between NIPs and NOD2 CD associated mutants could contribute to the pathophysiology and severity of CD.

**Fig 6:**
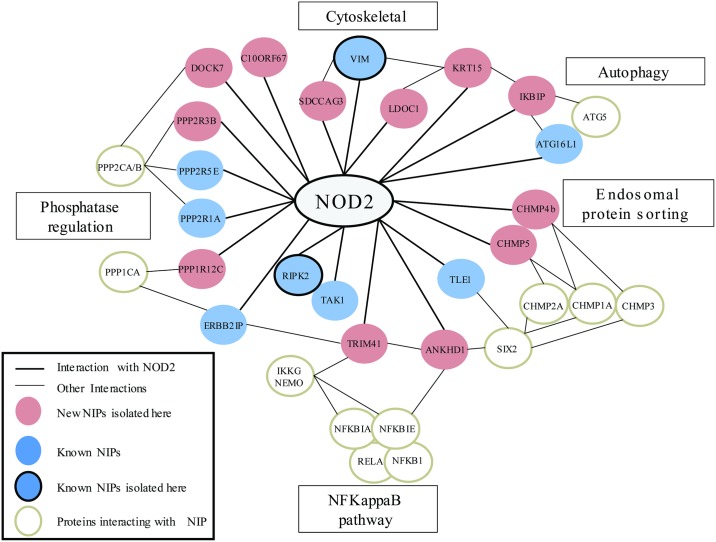
A NOD2 interactome network including new NIPs and possible functions: This partial network includes the new NIPs isolated in this study and some other known NOD2 partners issued from public protein/protein interaction databases (BIOGRID3.4, STRING) [69, 77]. For clarity, not all proteins involved in the NOD2 network were represented but only the new NIPs identified in this study and some NOD2 partners interacting with these new NIPs. Red filled circles represent new NIPs isolated here. Blue filled circles represent already known NIPs. Bold circles represent NIPs also characterized in other published studies.

In Table 6 we summarize and discuss likely functional links existing between new NIPs and NOD2 and illustrate in Fig 6 how these proteins could connect within the NOD2 protein interaction network.

**Table 6:** Summary of hypotheses linking a given NIP with NOD2, putative functions and appropriate references.

NIP	Summary of functions of NIPs characterized in this study and putative links with NOD2 functions
**RICK**	**NOD2 effector for NF-κB activation/already described NIP**
**ANKHD1**	**Interacts with IκBε a non-canonical regulator of NF-κB and with TRIM41 another NIP [**39, 58, 59**]. Homology with ANKRD17 a NOD2 partner involved in NOD mediated responses in epithelial and myeloid cells [**60**]. In drosophila, it is involved in the activation of the immune deficiency pathway triggered by bacterial challenge and interacts with IKKγ[**58**]. ANKHD1 localized at the basolateral membrane and in tight junctions, reminiscent of NOD2 subcellular localization in intestinal epithelial cells (**S1 Fig **and [**22**])**
**CHMP5**	**ESCRTIII proteins are required for late MVB formation and their fusion with the lysosome. Involved also in autophagy [**61**] CHMP4b is another ESCRTIII protein also identified in our colon Y2H screens. CHMP5 regulates negatively NF-κB during osteoclasts formation [**41**] whereas CHMP4b downregulates constitutive NF-κB [**62**]. In drosophila, CHMP5 orthologous protein *shrub* modulates the immune deficiency pathway triggered by bacterial challenge [**58**]. Additionally, MVB protein content includes K63 polyubiquitylated proteins, a non-degradative modification targeting RICK and IKKγ, essential to activate NF-κB downstream of NOD2 [**63–65**]. Finally, MDP altered the interaction between NOD2 and CHMP5 (this study) and endosomal transport being crucial for NOD2 stimulation and function [**66**], CHMP5 could have a role in NOD2 routing.**
**SDCCAG3**	**Belongs to the retromer protein family involved in the endosomal to plasma membrane trafficking [**67**] / interacts with VIM [**68, 69**]**
**LDOC1**	**Apoptosis and negative regulation of NF-κB [**40**]/ Interacts with KRT15 [**32, 69**]**
**TRIM41**	**Tripartite motif protein, E3 Ubiquitin Ligase structurally related to TRIM27 a known NOD2 partner regulating NOD2 proteasomal degradation[**26, 48**]/ Interacts with IKKγ/NEMO protein involved in NF-κB signaling [**70**], TRIM41 binds also to ERBB2IP a reported NOD2 partner important for membrane localization and affecting NF-κB signaling [**22, 71**]. Interacts also with ANKHD1 [**71**]**
**PPP1R12C**	**Regulatory subunit of phosphatase PP1. Involved in the negative regulation of NF-κB in astrocytes [**38**]**
**KRT15**	**Intermediate filament protein/interacts with the NIPs LDOC1 and IKBIP [**32, 72**]**
**C10ORF67**^**a**^	**Genetic link with CD and sarcoidosis? [**73**]**
**VIMENTIN**	**Intermediate filament protein, VIM is an already reported NOD2 and RICK binding protein [**20, 39**]. Role in scaffolding or targeting NOD2 signaling complexes to intracellular location? /Up-regulated by NF-κB and LPS/Interacts also with the NIPs SDCCAG3, KRT15 [**68, 69**]**
**DOCK7**	**GTP Exchange Factor of RAC1 [**47**]. Involved in membrane trafficking of NOD2?**
**PPP2R3B**	**Two other PP2 regulatory subunits, PPP2R1A and PPP2R5E bind also NOD2 [**19, 61**]. PPP2R1A is involved in inhibition of NOD2 induced autophagy [**61**] and is a positive regulator of IL8 secretion [**57**] whereas PP2R5E is involved in NOD2 dependent NF-κB activation [**74**]**
**IKBIP**	**IKBIP interacts with ATG16L1 and with ATG5 two proteins involved in autophagy and CD [**4, 69, 71**]. IKBIP interacts also with KRT15 [**72**]**
**PRR16**	**?**

The NIPs identified in this study are possible candidate genes for susceptibility to CD or UC, another IBD condition. We thus analysed the main NIPs in a cohort of more than 300 European IBD families. This cohort may appear limited when considering the very large cohorts used in GWAS to date, however the type of analysis is quite different and good power can be attained with a modest number of inferred meioses from the family data.

Among all the tested NIPs, we observed a positive association with some haplotypes of VIM in the CD family group carrying no *NOD2* mutations. Unfortunately, this result was not reproduced in the second family sample. Similarly, a weak association has been reported by Stevens et al. between VIM polymorphisms and CD but without firm validation [20], especially in the largest genetic study [17]. We thus conclude that VIM cannot be retained as a CD susceptibility gene to date. For all the other NIPs we failed to detect a significant association for any of the genetic markers after correction for multiple testing. Of note, the negative result obtained for *RICK*, a well-known NIP [75] was likely due to a lack of power. Indeed, RICK polymorphisms have been found associated with CD in the large GWAS meta-analysis published by Jostins et al. [17].

Among all the candidate genes analyzed here, *C10orf67* and *SDCCAG3* have been proposed as putative genes associated with CD [73, 76]. *C10orf67* was found following a genome scan to isolate genes associated with sarcoidosis and CD. The *SDCCAG3* locus is in a region where a SNP associated with CD was found in the vicinity of another IBD gene candidate, *CARD9*. However, none of the SNPs tested here for *SDCCAG3* and *C10orf67* showed any association. For *SDCCAG3* and *C10orf67*, we also genotyped the two SNPs previously found to be associated rs10870077 and rs1398024 respectively and we were unable to replicate the previous observation (for CD rs10870077 T:192; U:167 p = 0.19; rs1398024 T:140; U:122 p = 0.27). It is nevertheless very striking that the *C10orf67* locus was identified in our study on *NOD2* partners and in an independent study of CD genetics based on totally different screening procedure [73]. The authors claim that the susceptibility gene should be located within a region comprising *C10orf67* and *C10orf115*. Surprisingly the cDNAs sequences of our Y2H positives clones were actually chimeric between the *C10orf67* and *C10orf115* loci. However, the full cDNA that was used in this study for validation experiments spans only sequences of the *C10orf67* gene. Further biochemical studies are thus required to determine the exact nature of protein(s) encoded by transcripts arising from this 10p13 region and whether they interact with NOD2 and could possibly contribute to CD susceptibility. In summary, we report here a set of new NOD2 protein partners that connect into the NOD2 protein network (Fig 6). These data expand the definition of the NOD2 interactome and indicate that NOD2 may be involved in additional cell biological functions, including endosomal trafficking. Our results also suggest that the NOD2 protein network is influenced by NOD2 mutations found in CD. Further functional studies on this network of NOD2 proteins should help to delineate how NOD2 signaling protein complexes contribute to intestinal homeostasis and CD susceptibility.

## Supporting Information

S1 Fig ANKHD1 is expressed in intestinal mucosa. Immunofluorescence in intestinal mucosa using a purified rabbit polyclonal anti-peptide antibody recognizing ANKHD1. Fluorescence images (right) are shown in parallel with the Nomarski field (left). The white scale bar represents 10μm.
(PDF)

S1 Table Detailed phenotype for 59 selected Y2H clones (17 NIP candidates) identified in the lung library. Growing yeast colonies were tested and scored by 3 phenotypic assays (His+3-AT, Ura+, β-Gal).
(PDF)

S2 Table Additional NOD2 Y2H preys identified in the colon library. (PDF)

## Abbreviations

NF-κB: Nuclear Factor kappaB
MAPK: mitogen activated protein kinase
CD: Crohn’s Disease
IBD: Inflammatory bowel disease
Y2H: yeast two hybrid
NIP: NOD2 interacting protein
BRET: Bioluminescence resonance energy transfer
ESCRT: endosomal sorting complexes required for transport
MDP: muramyl dipeptide
LPS: Lipopolysaccharide
TDT: transmission disequilibrium test
TLR: Toll like receptors
NLR: NOD like receptors
IL-1β: Interleukin-1beta
GWAS: genome wide association studies
IL-10: interleukin-10
UC: ulcerative colitis
IC: intermediate colitis
SNP: single nucleotide polymorphism
3AT: 3-Amino-1,2,4-triazole
EGFP: enhanced green fluorescent protein
EYFP: enhanced yellow fluorescent protein
TX100: Triton X100
SC-Leu-Trp: minimal selective media lacking leucine and tryptophan
β-GAL: beta-galactosidase
Luc: Renilla Luciferase
MVB: multivesicular bodies
RLU: relative luciferase unit
