# Supplementary material for: Characterization and Genetic Analyses of New Genes Coding for NOD2 Interacting Proteins
Source: PLoS One. 2016 Nov 3;11(11):e0165420. doi: 10.1371/journal.pone.0165420 (PMC5094585; doi:10.1371/journal.pone.0165420)

S1 Fig: ANKHD1 is expressed in intestinal mucosa. Immunofluorescence in intestinal mucosa using a purified rabbit polyclonal anti-peptide antibody recognizing ANKHD1. Fluorescence images (right) are shown in parallel with the Nomarski field (left). The white scale bar represents 10µm.

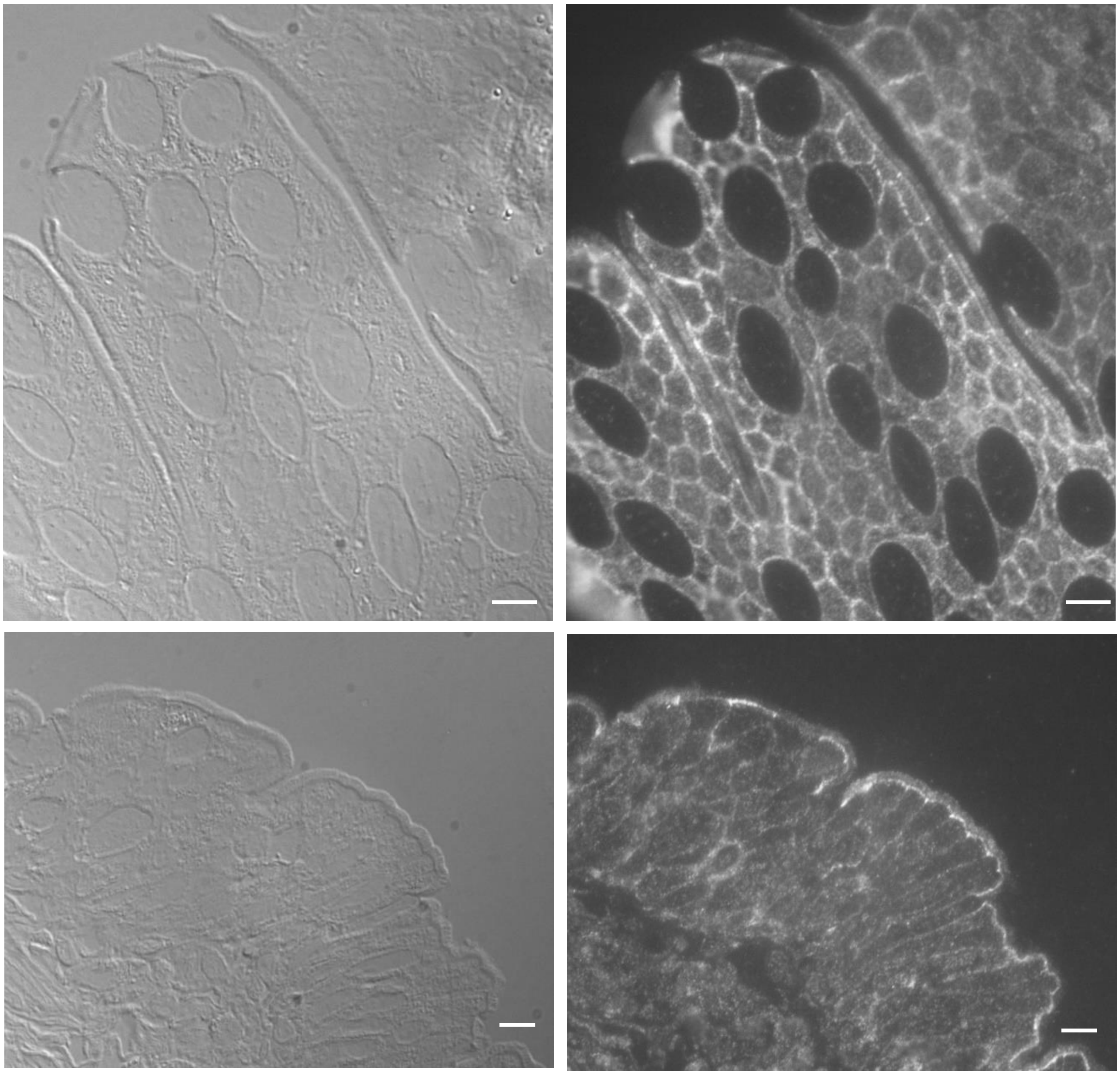

Supplement: S1 Fig — Immunofluorescence in intestinal mucosa using a purified rabbit polyclonal anti-peptide antibody recognizing ANKHD1. Fluorescence images (right) are shown in parallel with the Nomarski field (left). The white scale bar represents 10μm. (PDF) [file pone.0165420.s001.pdf]
