# Supplementary material for: Characterization and Genetic Analyses of New Genes Coding for NOD2 Interacting Proteins
Source: PLoS One. 2016 Nov 3;11(11):e0165420. doi: 10.1371/journal.pone.0165420 (PMC5094585; doi:10.1371/journal.pone.0165420)
Supplement: S1 Table — Growing yeast colonies were tested and scored by 3 phenotypic assays (His+3-AT, Ura+, β-Gal). (PDF) [file pone.0165420.s002.pdf]

S1 Table: Detailed phenotype for 59 selected Y2H clones (17 NIP candidates) identified in the lung library: Growing yeast colonies were tested and scored by 3 phenotypic assays (His+3-AT, Ura+, β-Gal).

Gene ID	HIS	URA	LACZ
**RICK**	**3+**	**2+**	**2+**
	**3+**	**3+**	**3+**
	**3+**	**1+**	**2+**
	**3+**	**NEG**	**2+**
**DOCK7**	**3+**	**NEG**	**3+**
	**3+**	**2+**	**2+**
	**3+**	**NEG**	**3+**
**DCTN1**	**3+**	**1+/-**	**3+**
	**3+**	**1+**	**3+**
**GOLGB1**	**2+**	**3+**	**3+**
	**3+**	**3+**	**3+**
**PRR16**	**2+**	**2+**	**3+**
**ANKHD1**	**3+**	**1+**	**3+**
**TRIM41**	**2+**	**NEG**	**2+**
	**2+**	**1+**	**1+**
	**3+**	**1+/-**	**2+**
	**2+**	**2+**	**NEG**
**CHMP5**	**2+**	**1+/-**	**2+**
	**2+**	**1+**	**1+**
	**3+**	**NEG**	**2+**
	**2+**	**2+**	**2+**
**SDCCAG3**	**2+**	**2+**	**NEG**
	**3+**	**2+**	**1+**
	**2+**	**2+**	**NEG**
**C10ORF67/ C10ORF115**	**3+**	**3+**	**3+**
	**3+**	**3+**	**3+**
	**3+**	**3+**	**3+**
	**3+**	**3+**	**3+**
	**3+**	**3+**	**3+**
	**3+**	**3+**	**3+**
	**3+**	**3+**	**3+**
**VIM**	**3+**	**2+**	**3+**
**IKBIP**	**3+**	**2+**	**3+**
	**3+**	**1+**	**2+**
	**3+**	**2+**	**3+**
	**3+**	**3+**	**3+**
	**3+**	**2+**	**3+**
**GOLGA6L5**	**3+**	**3+**	**3+**
	**3+**	**3+**	**3+**
	**3+**	**3+**	**3+**
	**3+**	**3+**	**3+**
	**3+**	**3+**	**3+**
	**3+**	**2+**	**3+**
	**3+**	**2+**	**3+**
	**3+**	**3+**	**3+**
	**3+**	**3+**	**3+**
	**3+**	**3+**	**3+**
**KRT15**	**3+**	**1+**	**3+**
	**3+**	**1+**	**2+**
	**3+**	**1+**	**2+**
	**3+**	**1+**	**2+**
**PPP1R12C**	**2+**	**2+**	**3+**
	**2+**	**2+**	**3+**
	**3+**	**3+**	**3+**
	**2+**	**2+**	**3+**
**LDOC1**	**3+**	**NEG**	**3+**
	**3+**	**NEG**	**3+**
	**3+**	**NEG**	**3+**
**PPP2R3B**	**3+**	**1+**	**3+**
