# Supplementary material for: Characterization and Genetic Analyses of New Genes Coding for NOD2 Interacting Proteins
Source: PLoS One. 2016 Nov 3;11(11):e0165420. doi: 10.1371/journal.pone.0165420 (PMC5094585; doi:10.1371/journal.pone.0165420)
Supplement: S2 Table — (PDF) [file pone.0165420.s003.pdf]

S2 Table: additional NOD2 Y2H preys fished out from colon library

Y2H preys isolated with NOD2 bait	Screen 1	Screen 2	Screen3
CHMP4b	6	7	3
PDLIM5Annexin A2	1	-	-
TACC3	1	-	-
HAP1	1	-	-
Annexin A2	-	1	-
C9ORF150	-	1	6
SCYL1	-	1	-
Tropomyosin alpha	-	1	-
NDKinase type 6	-	1	-
RPL13A	-	1	-
ALPI /Intestinal alkaline phosphatase	-	1	-
VCP/valosin-containing protein	-	-	2
lamin A	-	-	2
WBP11/ SNP70	-	-	2
TNIP1	-	-	1
